# Non-Invasive Imaging in Diabetic Cardiomyopathy

**DOI:** 10.3390/jcdd6020018

**Published:** 2019-04-16

**Authors:** Ify R. Mordi

**Affiliations:** Division of Molecular and Clinical Medicine, University of Dundee, Ninewells Hospital and Medical School, Dundee DD1 9SY, UK; i.mordi@dundee.ac.uk

**Keywords:** diabetic cardiomyopathy, heart failure, echocardiography, nuclear imaging, cardiac MRI

## Abstract

There is increasing recognition of a specific diabetic cardiomyopathy beyond ischemic cardiomyopathy, which leads to structural and functional myocardial abnormalities. The aim of this review is to summarize the recent literature on diagnostic findings and prognostic significance of non-invasive imaging including echocardiography, nuclear imaging, computed tomography and cardiovascular magnetic resonance in diabetic cardiomyopathy.

## 1. Introduction

The high incidence and prevalence of heart failure (HF) in individuals with type 2 diabetes (T2D) is well-established. Observational studies have shown an association between diabetes and risk of HF, as well as a link between worse glycaemic control and HF [1]. In diabetes trials the incidence of HF hospitalization was up to 13% [2]. Previously, it was commonly thought that T2D caused HF as a result of macrovascular disease (i.e., myocardial infarction) leading to HF with reduced ejection fraction (HFrEF). There is increasing recognition however of the existence of HF with preserved ejection fraction (HFpEF) as a distinct entity. HFpEF is typically associated with left ventricular hypertrophy (LVH), diastolic dysfunction and subclinical left ventricular systolic impairment. Many of these changes occur in individuals with T2D, and in the absence of other causes such as coronary artery disease (CAD), hypertension or valvular heart disease, this process can be described as diabetic cardiomyopathy (DCM) [3,4]. The pathophysiology of DCM is multi-factorial, but is thought to relate to the milieu of hyperglycaemia, hyperinsulinaemia and insulin resistance which leads to inflammation, oxidative stress, fibrosis, impaired myocyte metabolism and microvascular dysfunction.

Non-invasive imaging has the ability to provide important pathophysiological insights into the processes that lead to development of DCM. The aim of this review is to summarize the literature on the diagnostic and prognostic use of non-invasive imaging techniques in DCM.

## 2. Echocardiography

Measurement of left ventricular ejection fraction (LVEF) by echocardiography remains the first-line technique for evaluation of DCM. Diabetes is a highly prevalent comorbidity in patients with HF [5], independent of the presence of coronary artery or other causes of HF [6,7]. LVEF is as strongly associated with prognosis in T2D patients compared to those without T2D, and should be used for treatment decisions as per standard HF guidelines. Nevertheless, it is widely recognized that 2-dimensional assessment of LVEF has its limitations, and thus there is interest in other echocardiographic parameters in diagnosis and prognosis of DCM.

T2D has been strongly associated with adverse LV remodeling, most commonly concentric in nature. In several longitudinal cohort studies T2D has been independently associated with increased LV mass [8,9,10]. As in non-T2D populations, the presence of left ventricular hypertrophy (LVH) remains a marker of poor prognosis [11,12]. In the Framingham Offspring study of 4127 patients, T2D was independently associated with a more rapid increase in LV mass over the 16 year follow-up period [13]. Another large cohort study found that T2D was associated with LV dilatation and eventually a decline in LV systolic function over time [14]. There has also been some observational data suggesting that improvements in blood glucose control may be associated with reduction in LV mass, though this may be confounded [15]. There is limited randomized trial data on T2D therapies regressing LV mass in patients with T2D, although the EMPA-HEART trial (NCT02998970) did report some reduction in LV mass compared to the placebo. Extrapolating from hypertension data however, it is likely that regression of LVH by any pharmacological means is likely to improve outcome (though certain drug classes, such as angiotensin-converting enzyme inhibitors may be more likely to cause LV mass regression) [16,17].

Diastolic dysfunction has also been frequently characterized as one of the key echocardiographic findings in DCM. An early small study of 46 asymptomatic males with T2D found that diastolic dysfunction, defined as either abnormal relaxation or pseudonormalisation of left ventricular filling, assessed by transmitral pulse wave velocity imaging (E/A) and pulmonary venous recordings, was present in over half of the patients [18]. A larger study of 1760 T2D patients reported a diastolic dysfunction prevalence of 23% using tissue Doppler imaging (specifically E/e’ ratio > 15) [19]. In this study the presence of diastolic dysfunction was also associated with increased risk of death and subsequent HF. A more recent study also replicated this finding, reporting the prognostic significance of E/e’ ratio >15 in 406 T2D patients [20]. It is important to note however that diastolic dysfunction can also be caused by many comorbid conditions frequently found in T2D patients such as age, obesity and hypertension, and many of these studies did not take these into account when attempting to ascertain the independent nature of T2D for the development of diastolic dysfunction. Indeed, studies have reported that when these other variables are excluded, diastolic dysfunction may not be such an early component of DCM [21].

**Figure 1:**
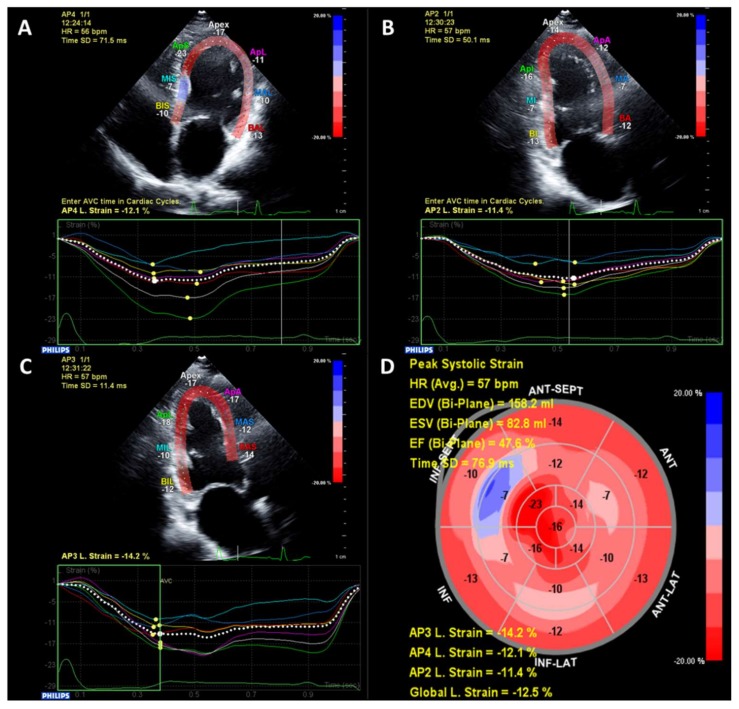
An example of echocardiographic speckle-tracking strain analysis. Images are taken in the apical 4-chamber (**A**), 2-chamber (**B**) and 3-chamber (**C**). A bullseye plot combining all three views is generated with reduced global longitudinal strain of −12.5% and reduced LVEF (47.6%) (**D**).

More recently, advanced echocardiography methods such as strain imaging using speckle-tracking have identified subclinical alterations in systolic myocardial function in patients with T2D and preserved LVEF (typically ≥50%) [22]. Strain is a measurement of myocardial deformation which is typically reported in three dimensions—longitudinal, circumferential and radial [23]. Strain is reported as a percentage change from diastole to systole, therefore due to the longitudinal and circumferential shortening of the myocardium lower values of longitudinal and circumferential strain represent better systolic function, whereas due to systolic myocardial thickening, higher values represent better systolic radial function. An example of strain imaging is shown in Figure 1. Global longitudinal strain (GLS) is the most studied parameter as it is more robust and reproducible, and also has independent, incremental risk prediction above and beyond ejection fraction [23,24]. GLS is frequently reduced even in T2D patients without overt structural heart disease compared to non-T2D patients [22]. Studies suggest that alterations in GLS may actually be a more sensitive marker of DCM than diastolic dysfunction [21].

GLS also appears to have prognostic significance. In one study of 172 asymptomatic patients with T2D, reductions in GLS at baseline have been associated with higher LV end-systolic volumes and LV relative wall thickness, as well as reduced likelihood of a decrease in LV volumes at three years [25]. Extending this further to hard outcomes, in a study of 230 asymptomatic T2D patients with LVEF ≥50%, almost half of the patients actually had subclinical evidence of systolic impairment with a reduction in GLS [26]. After 10 years of follow-up, GLS was independently associated with the primary endpoint of all-cause mortality and hospitalization, with a relative risk increase of 10% per 1% decrease in GLS. These results have been replicated in other studies [27].

Although the majority of studies have focused on the left ventricle, reduced left atrial GLS has also been reported in T2D patients [28,29]. Reductions in right atrial and ventricular GLS in T2D patients compared to controls have also been described [30]. No studies have specifically examined the prognostic significance of these parameters in T2D patients, however they do appear to have some association with outcome in HF population studies which have included T2D individuals [31,32,33].

There has also been some evidence that DCM is associated with alterations in left ventricular function during exercise or stress echocardiography. Similar to the general population, identification of epicardial CAD using stress echocardiography has prognostic significance [34,35]. There is now increasing recognition of the prevalence of coronary microvascular disease in patients with T2D, and stress echocardiography can be used to identify this. An initial study using tissue Doppler imaging did not find any significant stress-induced myocardial response in T2D patients undergoing dobutamine stress echo (DSE) compared to controls [36], however another study using exercise echocardiography (which may be more physiological) did find that T2D patients had abnormal longitudinal functional reserve [37]. Recent work has also suggested that the use of GLS during DSE may identify even more subtle early changes in asymptomatic T2D patients compared to controls [38].

Stress echocardiography may also provide some additional prognostic information. Measurement of coronary flow reserve (CFR), which in the absence of flow-limiting epicardial coronary artery stenosis is reflective of microvascular disease, was found to be associated with major adverse cardiovascular events in T2D patients with suspected angina and non-obstructed coronary arteries on invasive angiography [39]. Similarly, reduced CFR in the absence of resting wall motion abnormalities was also significantly associated with adverse outcome in asymptomatic patients [40].

Overall, echocardiography is the workhorse for clinical assessment of cardiac structure and function, and provides excellent temporal resolution for evaluation of subclinical LV dysfunction using strain and diastolic function. It has a wealth and depth of long-term prognostic information, and comprehensive echocardiography should certainly be considered as a key part of the evaluation of DCM.

## 3. Nuclear Imaging

The most common use of nuclear medicine in non-invasive CV imaging is for ischemia testing. Stress testing using single-positron emission computed tomography (SPECT) has been the most widely studied modality and has been shown to have similar diagnostic accuracy in T2D patients compared to non-T2D for detection of significant CAD compared to invasive angiography [41]. As with stress echocardiography, detection of ischemia in T2D patients is also associated with adverse prognosis [42]. In a study of 575 T2D patients with suspected angina referred for SPECT, the presence of any ischemia was associated with twice the risk of CV death or non-fatal myocardial infarction over a median of 4.4 years of follow-up (5.7% vs. 2.6%) [43]. This prognostic association is also apparent in asymptomatic T2D patients. In a large cohort of 1427 patients, a high-risk SPECT study (defined as summed stress score ≤47) was identified in 18% of the cohort and was significantly associated with mortality over a 10-year follow-up period, again with almost double the risk compared to those with low-risk studies (5.9% vs. 3.6%) [44]. Similar results have been reported in other smaller cohorts [45,46].

Alterations in CFR can also be detected by nuclear imaging, and similar to echocardiography, even asymptomatic T2D patients with normal coronary arteries demonstrate reduced CFR compared to controls measured during technetium sestamibi scanning [47]. Myocardial blood flow (MBF) and CFR can be more accurately quantified during PET (positron emission tomography) scanning. PET studies have also showed reductions in MBF during dipyridamole stress in asymptomatic T2D patients compared to controls [48]. Using rubidium-PET, Potier et al. studied 175 patients, without significant obstructive CAD, including 118 T2D patients, and found reduced myocardial flow reserved compared to non-T2D individuals [49]. Furthermore, the authors found a significant association with presence of albuminuria, suggesting that there may be a shared microvascular pathology. Prognostic data on PET is also available. In a large study, Murthy et al. evaluated 2783 consecutive patients (1172 T2D, 1611 non-T2D) and reported several findings [50]. First, similar to other modalities, T2D patients had significantly lower CFR and MBF. Second, while individuals with impaired CFR had significantly higher risk of death during follow-up regardless of T2D status, those with T2D had an even higher mortality risk. Indeed, T2D patients with impaired CFR had an outcome comparable to non-T2D patients with known CAD.

PET may also provide some useful information on other parameters. Rijzewijk et al. reported that T2D patients who underwent PET scanning displayed a reduction in myocardial glucose uptake and increases in fatty acid uptake and oxidation, although these were not correlated with LV diastolic function [51]. A recent study also using PET also reported that T2D was associated with decreased myocardial glucose metabolism [52].

Nuclear imaging remains widely used, particularly in ischemia assessment where its diagnostic and prognostic value has been extensively validated. There is no evidence to suggest that the prognostic value of nuclear imaging is any different in T2D patients compared to non-T2D. Nuclear imaging also allows assessment of myocardial blood flow, and some assessment of metabolism, which might provide further pathophysiological insights into DCM.

## 4. Cardiovascular Computed Tomography

The main use of cardiac computed tomography (CCT) is for non-invasive visualization of coronary arteries. CCT offers unparalleled anatomical delineation of cardiac structures compared to other non-invasive imaging methods. The majority of CCT studies have focused more on identification of CAD than DCM, however CCT may play an important role in confirming DCM by excluding significant CAD as a cause for HF. As in non-T2D populations, the presence of significant obstructive CAD is associated with adverse cardiovascular outcomes. In asymptomatic individuals, a large meta-analysis of 10 studies including 5012 individuals with T2D reported that the presence of both obstructive CAD (≥50% stenosis) and non-obstructive were significantly associated with adverse events, as was the extent of CAD [53]. T2D patients also have higher calcium scores than non-T2D individuals, and again, this is associated with adverse prognosis [54].

Beyond assessment of the coronary arteries however, there have been few large studies assessing the role of CCT in DCM. It is possible to measure ventricular function using CCT, although given the ionizing radiation dose, other methods such as echocardiography are preferred [55]. Perfusion imaging can also be performed using CCT, although it is not routinely used in clinical practice, again due to the increased radiation dose required to conduct the study. As with other modalities, T2D patients with suspected CAD but no obvious perfusion defect displayed reduced MBF compared to non-T2D individuals [56]. Reduced MBF may be associated with longer duration of diabetes [57].

The main role of CCT in DCM is in providing a non-invasive method to exclude significant CAD as the underlying aetiology. Given that most guidelines do suggest that high-risk patients such as those with T2D should undergo invasive coronary artery assessment, CCT does not play a huge role in assessment of DCM. It may, however, be valuable in those unable to undergo invasive coronary angiography or cardiac magnetic resonance imaging, or in those whose echocardiographic images are sub-optimal.

## 5. Cardiovascular Magnetic Resonance

Cardiovascular magnetic resonance imaging (CMR) is becoming increasingly utilized to provide further pathophysiological insight into myocardial structure and function. As well as being the gold-standard for measurement of left ventricular volumes, function and mass, CMR also has unique tissue characterization properties which have helped shed light on the patterns and prevalence of DCM, some of which have also been shown to be prognostically significant.

The most widely studied CMR technique is that of late gadolinium enhancement (LGE). This technique is usually performed by taking CMR images 10–15 min after the injection of intravenous gadolinium contrast agents. Gadolinium is an extracellular contrast agent, and therefore in normal healthy myocardium where there is very little extracellular space, no gadolinium remains, and the myocardium appears black. In areas where there is extracellular space (e.g., infarct, fibrosis, scarring), the myocardium appears white due to the gadolinium contrast [58]. The presence of an infarct pattern of LGE can predict unrecognized, “silent” myocardial infarction in patients with diabetes and is associated with worse cardiovascular outcome [59]. This may herald underlying significant CAD and hence may prompt changes in preventative therapies as well as invasive coronary angiography.

LGE techniques are predominantly only able to identify focal areas of fibrosis. More recently, there has been intense interest in measuring diffuse myocardial fibrosis using T1 mapping techniques [60]. T1 mapping provides a quantitative measure of the myocardial T1 relaxation time and can be performed without contrast (native) or post-gadolinium contrast (allowing calculation of myocardial extracellular volume, ECV). T1 mapping techniques can differentiate between patients with cardiomyopathy and healthy controls independent of ejection fraction and are also related to exercise capacity, subclinical LV dysfunction and prognosis [61,62,63]. In patients with T2D without any LGE, increased interstitial fibrosis (shorter post-contrast T1 time) was significantly associated with GLS and diastolic dysfunction [64]. In another CMR study, asymptomatic T2D patients with microalbuminuria had higher ECV and high-sensitivity troponin as well as diastolic dysfunction [65]. Although there is no specific prognostic data for T1 mapping or ECV in T2D patients, given that T2D patients have higher ECV than controls it is likely that a similar prognostic association would be seen as in the general population.

Magnetic resonance spectroscopy (MRS) imaging has also been utilized to demonstrate increased myocardial triglyceride content in T2D using proton (^1^H) MRS, reflecting cardiac steatosis which is present even in individuals with preserved LVEF [66]. This steatosis is associated with reductions in systolic strain and concentric LV remodeling that are features of DCM as well as impaired myocardial energetics measured using ^31^P (phosphorus) MRS [67]. Indeed, measurement of the myocardial phosphocreatinine to adenosine triphosphate ratio (PCr/ATP) using ^31^P MRS is a non-invasive measure of myocardial energetics which is reduced in individuals with T2D, correlates with impaired perfusion and oxygenation, coronary microvascular dysfunction, diastolic dysfunction and subclinical systolic impairment [68].

CMR has also been used to identify epicardial adipose tissue (EAT), which is associated with T2D and insulin resistance. EAT has been associated with reduced systolic strain in DCM [69] and peripheral arterial stiffness [70], and thus may play an important pathophysiological role in DCM. Finally, stress perfusion CMR can also be used to identify T2D patients with suspected ischemia in a similar manner to MPS or DSE [71], but also identifies microvascular ischemia in the absence of significant epicardial CAD [72].

## 6. Future Directions

In this review the main non-invasive imaging techniques used in clinical practice have been summarized. Nevertheless, many of the advanced techniques discussed such as speckle-tracking echocardiography, PET and cardiovascular magnetic resonance are not routine used by clinicians and are limited to research. It is likely, as more prognostic data becomes available, that these techniques are used more in the future. In particular, some of the changes identified in DCM using these techniques (such as altered MBF, myocardial substrate utilization and energetics) may become treatment targets and require monitoring using advanced non-invasive imaging. With the increasing interest in CMR T1 mapping techniques, there will almost certainly be prognostic data on diffuse myocardial fibrosis in DCM, and whether therapies can be developed to reduce this fibrosis. There is also increasing focus on combining imaging techniques, either in cluster analysis (to identify patients with similar phenotypes who might have differing prognosis and respond to treatment differently) [73] or alongside biomarkers [74]. As novel imaging techniques continue to be refined and more clinical data becomes available, non-invasive imaging will continue to provide valuable insights into DCM.

## 7. Conclusions

**Figure 2:**
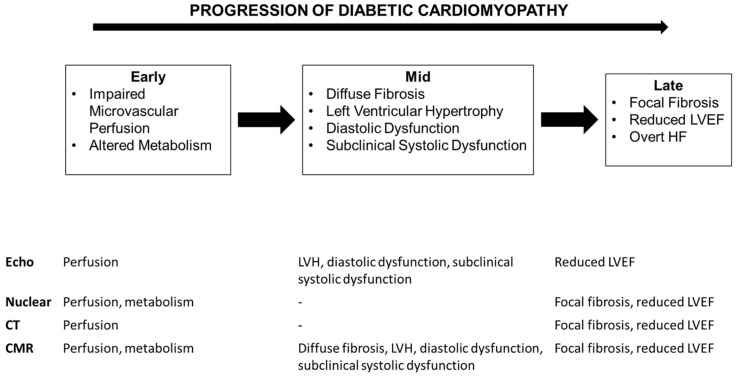
Overview of the structural and functional abnormalities occurring in the progression of diabetic cardiomyopathy and the non-invasive imaging techniques that can be used to assess these.

T2D patients are at significantly increased risk of CV events compared to the general population. Beyond CAD, T2D itself appears to be associated with a number of myocardial structural and functional changes leading to DCM and HF. Non-invasive imaging techniques can play a key role in identification of these changes even prior to the development of overt HF [75]. Figure 2 summarizes the various myocardial changes seen in DCM and the non-invasive imaging techniques that can be used for their identification. Larger studies are now needed to determine the prognostic significance of these newer techniques in addition to traditional risk markers, and intervention trials to determine whether targeting these imaging parameters improves CV outcome.
